# Evaluation of Quality and Storability of “Italia” Table Grapes Kept on the Vine in Comparison to Cold Storage Techniques

**DOI:** 10.3390/foods10050943

**Published:** 2021-04-26

**Authors:** Francesca Piazzolla, Maria Luisa Amodio, Sandra Pati, Giancarlo Colelli

**Affiliations:** Dipartimento di Scienze Agrarie, Alimenti, Risorse Naturali e Ingegneria (DAFNE), Università di Foggia, Via Napoli 25, 71122 Foggia, Italy; francesca.piazzolla@unifg.it (F.P.); marialuisa.amodio@unifg.it (M.L.A.); sandra.pati@unifg.it (S.P.)

**Keywords:** modified atmosphere, carbon dioxide, phenols, antioxidant, ethanol, acetaldehyde

## Abstract

The aim of the study was to compare the quality of table grapes (cv. Italia) held on the vine compared to grapes stored in cold rooms with or without modified-atmosphere packaging (MAP). The grapes were harvested from 12 plants in 2 vineyards in the same area, differing for the age of the plant. Four- and a fourteen-year-old vines were cultivated with the “Apulia tendone” system. After the first harvest, grapes were divided into small clusters and used for storage treatments in air and in MAP. Samples of 400 g were packaged in polypropylene (PP) trays sealed with a polypropylene/polyamide (PP/PA) film with 20% CO_2_ in air. MAP and control samples were then stored in the same cold room at 0 °C. Initially and after 8, 21, and 28 days, grapes stored in air and MAP were compared to fresh harvested grapes, stored on the plants. Quality attributes included color, texture, maturity index, phenols, antioxidant activity, sugars, organic acids, sensory parameters, and volatile compounds. The results obtained demonstrated that grapes held on the plant and in MAP showed better quality in terms of appearance scores compared to grapes stored in air. In particular, the application of high CO_2_ contributed to reduce the deterioration rate of the clusters, minimizing weight loss, and delaying degradation processes, and this particularly for grapes from the 14-year-old vine, where grapes held on the plant degraded faster than grapes in the younger vines. Most volatile compounds did not change their concentration with the storage treatment, except for ethyl acetate and ethanol, which increased in MAP at the end of storage, and to some compound responsible for green odor. In conclusion, keeping the grapes on the plant can be considered a good agronomic practice to preserve the quality, whereas MAP can be applied to better maintain postharvest quality of the product throughout storage and distribution.

## 1. Introduction

Table grapes are one of the most consumed fruits in the world, and a valuable source of phytonutrients [1,2].

It is a non-climacteric fruit, and for this reason, unlike many other fruit crops, the ripening process does not continue off the vine. Sugars produced by photosynthesis are translocated from leaves to the grapes only until they are on the vine or until the maximum Brix degree is reached. Also, there is no conversion of starch into sugars, as the amount of starch in berries is very low [3]. Though the concentration of berry solutes can change after harvest, and some other quality-related compounds can evolve or be degraded, in general, grapes should be harvested only when the target quality parameters have been reached.

A common practice used in southern Italy is to appositely cover canopies with plastic film (i.e., low-density polyethylene) during August to delay the harvest times of table grapes from October to the late November or early December [4]. This “late” forcing, conversely to the “early” one, aimed to anticipate the shoot sprouting, is executed to delay the harvest as much as possible, [5] while protecting fruit form autumnal rains. In addition, forced “storage” has the main advantages of maintaining green and hydrated stalks, and, above all, to allow the use of fungicides, which cannot be applied during storage. Some authors [5,6] reported the benefits of this agronomic technique to meet the demand for high-quality table grapes during Christmas time, resulting in a higher profitability for the producers.

Piazzolla et al. [6], in fact, demonstrated that “late forcing” on the vine is a feasible approach to preserve and even improve table grape quality related to sensorial aspects, even if the authors reported that extreme delay could reduce the quality of the fruit. Nonetheless, nothing is known about quality and storability of “on-vine-stored” grapes and on the potential of this method compared to conventional postharvest storage techniques.

Table grapes are not chilling-sensitive [7]; the respiration rate and the rate of ethylene production for grapes are very low (2 mg CO_2_·kg^−1^·h^−1^ at 0 °C and less than 0.1 μL·kg·h^−1^ at 20 °C, respectively). Table grape quality is reduced by the occurrence of stem browning and *Botrytis cinerea* infections [8]. Recently, the main physical, chemical and bio-based treatments in postharvest for the control of *Botrytis cinerea* on table grapes have been reviewed by De Simone et al. [9]. Ideally, grapes are stored in cold rooms operating at −1 to 0 °C with 95% RH, and a very low airflow; in these conditions, grapes may be stored up to 4 weeks [10], but generally SO_2_ fumigation are also applied to better control the mold growth [11,12]. Standard commercial practices include initial sulfur dioxide (SO_2_) fumigation during pre-cooling, followed by weekly fumigations with similar doses during cold storage, or most commonly, SO_2_ generator pads inside boxes are widely used for table grape storage and transport [13]. As alternative, chitosan, and aloe vera gel treatments have been successfully proposed to maintain table grape quality and extend their shelf-life [14,15,16].

Other methods used to extend the storability of table grapes include controlled (CA) or modified atmospheres (MA), and active packaging.

As for CA, the addition of CO_2_ (10 to 15 KPa in air) can be effective in controlling grey mold (*Botrytis cinerea*) for 2 to 4 weeks depending on cultivars [7].

Crisosto et al. [17] concluded that the CA treatment with 10 kPa CO_2_ combined with O_2_ levels from 3 to 12 kPa limited grey mold infection on “Red globe” grapes during 12-weeks of cold storage, whereas early harvested “Red globe” could be stored only for 4 weeks in 10 kPa CO_2_ + 6 kPa O_2_. In addition, CA treatment [18] and low temperature storage combined with SO_2_ slow-release generators [19] may be effective as insecticidal control.

As for MAP, Artés-Hernández et al. [20], reported that “Autumn seedless” grapes stored for 2 months in MAP with 15 kPa O_2_ and 10 kPa CO_2_ at 0 °C followed by 1 week at 15 °C in air, helped to prevent rachis browning and flavor losses.

Costa et al. [21] selected the best packaging material to achieve at the equilibrium the desired gas conditions (10–15% O_2_ and 4% CO_2_) that could control the respiration, the water loss, and the rachis color changes of the grapes. Among the tested polymeric material, oriented polypropylene (80 μm of thickness) could allow a shelf-life longer than 70 days.

Silva-Sanzana et al. [22] reported that modified-atmosphere packaging controlled the green color losses on stalks of “Red Globe” grapes stored for 90 days at 0 °C, compared with a conventional storage even after a shelf-life period, but no comparison is available with grapes “stored on the vine”.

Cefola et al. [23] reported that the storage of “Italia” table grapes at high CO_2_ atmosphere (20 KPa) induced the shift to anaerobic metabolism, reporting an increase in respiration rate, and acetaldehyde and ethanol production, and a lower evaluation at the sensorial test, than grapes stored with lower CO_2_ concentrations (up to 10%) or in air. Finally, the use of an active packaging based on PET coated with a layered double Hydroxide (LDH) hosting 2-acetoxybenzoic anion (salicylate) as antimicrobial molecule was shown to cause a significant reduction in total mesophilic aerobic count and mold and yeast population with respect to control [24].

This work aimed to assess for the first time the quality and storability of table grapes stored on the vine, also considering quality evolution of grapes from the same plants stored in cold rooms (with or without modified-atmosphere packaging).

## 2. Material and Methods

### 2.1. Plant Material and Sample Preparation

“Italia” table grapes were grown in Foggia (Puglia, Italy, 41°28′39.2′′ N), in 2 contiguous vineyards of the same grower, but with plants of 2 different ages, 4 and 14 years. The vegetative system consisted of the “tendone” which was covered with a plastic net and a plastic film of LDPE. Grapes were harvested randomly from 12 marked plants in October, when according to the grower procedure, the commercial maturity was reached. Grapes were rapidly transported to the Postharvest Laboratory of the University of Foggia where clusters were divided into smaller clusters. Three replicates of 400 g were used for initial quality determinations while 18 samples of the same size were prepared for storage in air in macroperforated bags (AIR) or in modified-atmosphere packaging (MAP). For MAP, 9 samples were packaged in polypropylene (PP) trays sealed with a PP/PA film (polypropylene/polyamide, 30 µm, with CO_2_ transmission rate of 48 mL·m^−2^ day and O_2_ transmission rate of 135 mL·m^−2^ day) in active modified-atmosphere (20% CO_2_ in air) using a semi-automatic tabletop tray sealer (ILPRA termosaldatrici, FoodPack, Vigevano, PV, Italy). For AIR treatment the trays were wrapped in macroperforated bags containing wet paper pads to keep a high-RH environment around the product. Three replicate trays were prepared for each treatment and sampling time, with a total of 18 trays. All samples were stored at 0 °C for 28 days. After 8, 21, and 28 days of storage, 3 trays for each treatment (MAP and AIR) were used for quality determinations together with samples freshly harvested from the 12 marked plants (PLANT).

### 2.2. Quality Determination

The following quality parameters were analyzed initially, and on each sampling date.

Weight loss, berry firmness, gas concentration (for MAP samples only), visual quality (appearance score), color parameters (Hue Angle, Chroma) and sensorial quality (aroma, flavor, crunchiness, sweetness, acidity, fizzy taste, resistance to berry detachment, and overall evaluation) were monitored on fresh samples. About 100 g of berries were then frozen and stored at −80 °C until analysis with TSS, pH, TA, volatile compounds, organic acid, and sugar composition measured on the squeezed juice, whereas total phenol content and antioxidant activity were extracted from the skin. Additionally, at each sampling date ethanol and acetaldehyde extracts were prepared and frozen at −20 °C.

#### 2.2.1. Headspace Gas Determination and Weight Loss

Before opening the packages, the gas composition was determined using a gas analyzer with an accuracy of 0.5% for both O_2_ and CO_2_ (Witt, Gascontrol 100, MAPY4.0, Witten, Germany), equipped with an aspiration pump and a needle. A gas volume of 0.5 mL was automatically withdrawn and used for the gas analysis.

Weight loss was calculated as percentage of difference from initial weight.

#### 2.2.2. Color Analysis

Color indexes were extracted from hyperspectral images acquired with a Spectral scanner (DV SRL, Padova, Italy), in the VisNir range (400–1000 nm, resolution 5 nm) as described in Piazzolla et al. [6]. From the primary L*, a*, and b*, Hue angle, and Chroma were calculated.

#### 2.2.3. Sensory Evaluation

Sensory attributes of the berries were scored by a 5-judges trained panel. The judges evaluated 3 berries with pedicel from each sampling replicate for each harvest time. Judges evaluated the aroma, flavor, crunchiness, sweetness, sourness, fizzy taste, resistance to berry detachment and the overall evaluation using a scale of 5 to 1, where 5 = very high; 3 = fair; 1 = very low.

#### 2.2.4. Firmness

Firmness was evaluated on 45 berries per replicate by a compression test, performed with an Instron Universal Testing Machine (model 3343, Norwood, MA, US), at a speed of 50 mm·min^−1^. The force (N) required for a 3 mm compression between two parallel plates (diameter of 10 cm) was recorded.

#### 2.2.5. Total Soluble Solids, pH, and Titatrable Acidity

Initially and for each sampling date, total soluble solids (TSS), pH and titratable acidity (TA) were assessed using 4 g of juice sample from 15 berries, for each replicate. TSS were measured using a digital refractometer (PR-32 Palette, Atago, Tokyo, Japan), while pH and TA were assessed with an automatic titrator (TitroMatic 1S, Crison, Toledo, Spain), titrating to pH 8.1; the value was expressed as percentage of tartaric acid.

#### 2.2.6. Total Phenolic Content and Antioxidant Activity

Total phenol content and antioxidant activity were determined on frozen samples. One gram of skin was added of 3 mL g^−1^ of methanol plus 3% formic acid and was homogenized with an Ultraturrax (IKA T18 basic, Wilmington, NC, USA) [25]. The extract was then centrifuged at 5 °C and 9000 rpm for 10 min. Total phenols were determined according to the method of Singleton and Rossi [26] and expressed as grams of gallic acid per kilogram of fresh weight (g GA·kg^−1^). Antioxidant assay was performed following the procedure described by Brand-Williams et al. [27] and reported in grams of Trolox equivalents per kilogram of fresh weight (g TE·kg^−1^).

#### 2.2.7. Simultaneous Analysis of Organic Acids and Sugars

All samples were thawed and then the juice from each sample was filtered with a C_18_ Sep-Pak cartridge (Grace Pure ^TM^, New York, NY, USA) and then with a 0.2 μm filter. After dilution (1:1) with ultrapure water, 10 μL-samples were injected into an HPLC system (Agilent 1200 series) equipped with an UV detector, set at 210 nm, and a refractive index detector. Compounds were separated on a Rezex ROA-Organic Acid H + (8%) column (300 × 7.80 mm) (Phenomenex, Torrance, CA, USA), using an aqueous mobile phase of 0.1% phosphoric acid, (flow rate of 0.5 mL·min^−1^) and an oven temperature of 30 °C. The different organic acids and sugars were quantified by chromatographic comparison with analytical standards. Resulted were expressed as g·kg^−1^.

#### 2.2.8. Determination of Ethanol and Acetaldehyde

For ethanol and acetaldehyde, 5 g of fresh table grape tissues were homogenized with 10 mL of water and then centrifuged at 5 °C and 9000 rpm for 10 min. Five mL of supernatant were placed into 20 mL glass vials and stored at −20 °C until analysis according to the method of Mateos et al. [28]. After thawing for 1 h in a water bath at 65 °C, 1 mL headspace gas sample was withdrawn and injected into a gas chromatograph (Shimadzu GC-14A, Tokyo, Japan) equipped with a FID detector (150 °C). Ethanol and acetaldehyde were identified by co-chromatography with standards and quantified by a calibration curve.

#### 2.2.9. Headspace Solid-Phase Microextraction (HS-SPME) and Gas-Chromatography Mass Spectrometry (GC-MS) Analysis

The extraction of volatile compounds was carried out by HS-SPME using an 85 μm Carboxen/Polydimethylsiloxane fibre (Supelco, Bellefonte, PA, USA) and a GC-MS instrument, according to Piazzolla et al. [6].

After thawing the fruit, and detaching seeds and pedicels, 100 g of fruit tissue were added with 2 g of CaCl_2_, 20 g of NaCl, 100 μL of internal standard solution (100 ppm 2-methyl pentanol methanolic solution) and homogenized using a commercial blender. The homogenized (8 g) was placed into a 15 mL capped SPME vial and stirred for 20 min, at 40 °C. Then, the fibre was exposed for 30 min to the capped vial headspace, manually injected into the GC (splitless mode) and kept for 4 min to allow for desorption of volatile compounds. The separation was achieved on a DB-WAX capillary column (60 m × 250 μm × 0.25 μm, J&W Scientific Inc., Folsom, CA, USA) and the identification by comparison of retention time and mass spectra with pure compounds when available, or with data system library (NIST 02, *p* > 80). All compound concentrations were expressed as µg of 2-methyl pentanol equivalent g^−1^.

#### 2.2.10. Statistical Analysis

For each vineyard, a 2-way factorial design for treatment (Air, AM, and PLANT) and time of storage (8, 21 and 28 days) was conducted. At each storage time, a 1-way ANOVA for the treatment was performed. Mean values were separated with Tukey test (*p* < 0.05).

The data were analyzed with StatGraphics Centurion software (ver.16.1.11, StatPoint Technologies, Inc., The Plains, VA, USA).

## 3. Results and Discussion

In Table 1 is reported, for each vineyard, the effect of the treatment and time of storage on quality attributes. Data of both vineyards were enough in agreement, and as will be better explained with data discussion, main differences were due to the higher quality of grapes from 4-year old vines, which degraded much slower, in comparison to the 14-year old grapes, particularly when stored in air, or kept on the plant. Treatment and time of storage affected more parameters for grapes from 14-year-old vines, than in the case of grapes from the 4-year-old vines. For grapes from 14-year-old vines, treatment influenced firmness, weight loss, hue angle, chroma, titratable acidity, phenol content, antioxidant activity, acetaldehyde, ethanol, citric acid and all sensorial parameters (except for sweetness). The time of storage affected most of parameters except for weight loss, phenols, antioxidant activity, fumaric acid, and fizzy taste score. On the other side, for grapes of 4-year-old vines, hue angle, acidity, phenols, were not affected by treatment and much less parameters were affected by time of storage. Since interaction between treatment and time of storage, was often significant when treatment was significant, and mostly for sensorial score, the simple effect of treatment for each quality attribute was evaluated at each sampling time.

As for gas evolution within packages, CO_2_ concentration was reduced for both experiment to 10% after 20 days of storage and remained constant until the end storage, while O_2_ stayed up to the atmospheric level (18–20%). After 28 days of storage, the CO_2_ and O_2_ concentrations were approximately 10.5 and 20%, respectively.

**Figure 1:**
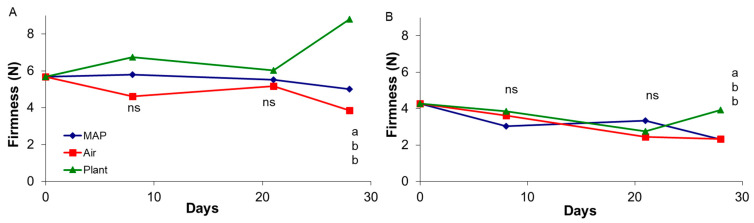
Effect of storage treatment on firmness of “Italia” table grape from a 4-year (**A**) and 14-year-old (**B**) vineyard during storage. At each sampling time different letters indicate mean values significantly different (*p* < 0.05 and Tukey test; ns: not significant).

In Figure 1 is shown the effect of treatment on firmness during the storage; in particular firmness of berries held in MAP remained practically unchanged until the end of storage for both experiments, when grapes hold on the PLANT showed higher firmness values, compared to grapes stored in air and in MAP. For grapes of 4-year-old vines, a singular increase of firmness values was observed on berries left on the plant for 28 days, suggesting a concentration of pectin and cellulose when there was less competition for nutrients between fruit. As for berry stored in AIR weight loss increased to 3.7 and 2.2% at the end of the storage, for grapes of 4- and 14-year-old vines, respectively.

**Figure 2:**
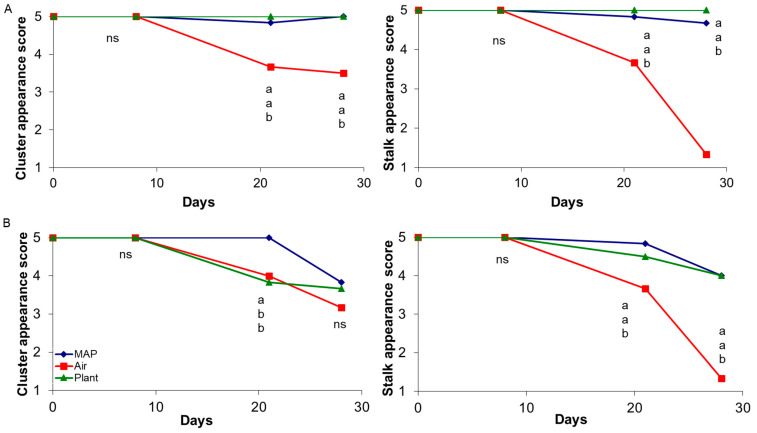
Effect of treatment on stalk and cluster appearance scores of “Italia” table grapes from a 4-year (**A**) and 14-year-old (**B**) vineyard during storage. At each sampling time different letters indicate mean values significantly different (*p* < 0.05 and Tukey test; ns: not significant).

Figure 2 shows the effect of treatment on cluster and stalk appearance scores during storage. Here the main difference due to the age of the plant can be observed. Although for 4-year-old vines, there was no difference over storage time for cluster appearance of grapes stored on the plant and in MAP, for grapes from 14-year-old vines, cluster score was best preserved in MAP up to 21 days of storage, with no difference after 28 days. In this case, grapes stored on the vine degraded much faster when hold on the plant or in the cold room, whereas the presence of CO_2_ was effective on delaying ethylene effects [29,30] for grapes from both vineyards.

In terms of visual appearance, the main effect of the different storage treatment was observed on the stalk, which showed dehydration and discoloration, for grapes stored in AIR, while less differences were observed for the berries, particularly for those from 14-year-old vines. Grape berries were less influenced by water loss, since they are well protected with waxy layers.

Stalk appearance showed a severe deterioration for grapes stored in AIR, whereas appearance scores remained almost unchanged for PLANT and MAP stored grapes. The score for AIR samples reached the value of 1 at 28 days, certainly for the observed dehydration, which occurred despite the protected conditions (macroperforated bag and humidified water pad). On the other hand, the high score values registered for samples in MAP are certainly related to low levels of dehydration but also to the effect of high-CO_2_ atmospheres on slowing down chlorophyll degradation rate. The effect of atmospheres with 29 kPa CO_2_ and 1 kPa O_2_ on chlorophyll retention has been demonstrated by Pariasca et al. [29] on pea pods and by Cefola et al. [31] on broccoli raabs stored with 10% CO_2_. Also, Silva-Sanzana et al. [22] reported that modified-atmosphere packaging helped to maintain green color of the stalk for “Red Globe” grapes stored for 90 days at 0 °C, with no negative effect on the quality of the berries. Similar results were found in “Autumn seedless” table grapes [20], indicating that clusters stored in air showed extreme browning of the stalk while clusters stored in CA (5 kPa O_2_+ 15 kPa CO_2_) and MAP (15 kPa O_2_+ 10 kPa CO_2_) had a good visual appearance at 60 days of storage at 0 °C and after additional 7 days in air at 15 °C.

**Figure 3:**
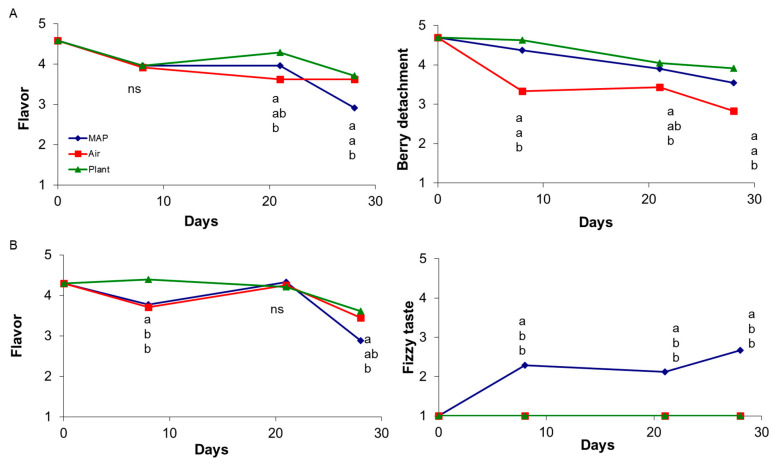
Effect of treatment on flavor and berry detachment over storage of “Italia” table grapes from a 4-year vineyard (**A**) and on flavor and fizzy taste on grapes from 14-year-old (**B**) vineyard. At each sampling time different letters indicate mean values significantly different (*p* < 0.05 and Tukey test; ns: not significant).

Berry resistance to detachment decreased over time for all treatments; grapes stored in AIR showed a lower resistance to berry detachment than grapes hold on the PLANT or in MAP for grapes from 4-year old vines (Figure 3), and up to 21 days of storage for grapes from 14-year-old vine, where at 28 days PLANT showed highest value (3.7 N) and both air and MAP the same value of about 2.4 N. These results can be in part attributed to the different degree of water loss of stalk suffered by samples of different treatments. Dehydration stress, in fact stimulates ethylene production which in turn favors the formation of the abscission layer, which reduces the force required for berry detachment. Pariasca et al. [29] and Bailén [30], found that the CO_2_ inhibits the abscission layer in MAP samples, because of the known competition of CO_2_ with ethylene on binding sites. In this case, for grapes of 14-year-old vines CO_2_ could had prevented ethylene effects, up to 21 days, but at the end of the storage in both MAP and AIR samples senescence processes were not inhibited, and no differences in berry detachment were observed.

**Figure 4:**
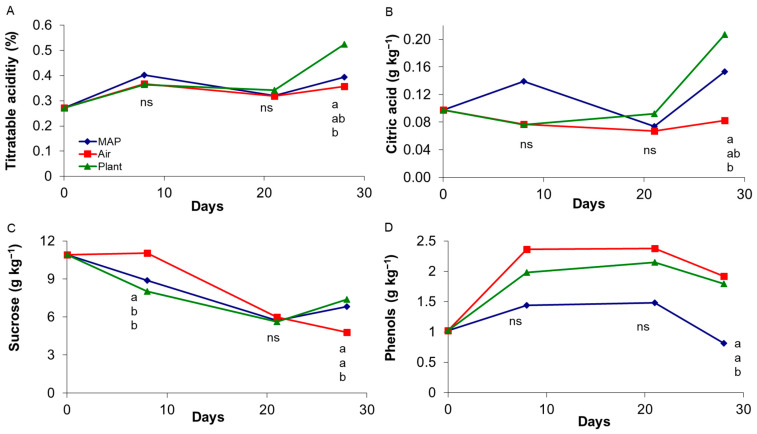
Effect of storage treatment on titratable acidity (**A**), citric acid (**B**), sucrose (**C**) and phenols (**D**) of “Italia” table grapes from a 14-year-old vineyard during storage. At each sampling time different letters indicate mean values significantly different (*p* < 0.05 and Tukey test; ns: not significant).

As for the difference in color observed particularly for grapes of the oldest vine, a slightly higher decrease of b*, Chroma (also for the 4-year-old-vine), and Hue angle values was observed for grapes stored on the plant, which also showed a higher increase of the a* value. This may be explained by a higher enzymatic activity for grapes stored on the PLANT, and on the same time by the reduction of photosynthetic activity. Nonetheless difference in color were very minimum and not perceived by eyes. Regarding chemical attributes: titratable acidity, phenols, citric acid, and sucrose were significantly affected by the treatment, showing the same trend for grapes from both vineyards (data for grapes of 14-year-old vines is shown in Figure 4).

Titratable acidity and citric acid content increased after 28 days of storage for grapes store on the PLANT, which presented a higher content than AIR, while intermediate values were observed for MAP. Probably, the reduction of the fruit load could have led to a stimulation of the vegetative activity of the plant, inducing the increase of the acidity in the fruit. No differences for the other individual organic acids were found; tartaric and malic acids were the most abundant acids, followed by citric and succinic. In addition, traces of fumaric acid were also detected, in agreement with what reported in the literature for other varieties [32,33].

On the other hand, sucrose content decreased during storage; after 28 days, grapes stored in AIR showed a lower content (4.8 g·kg^−1^) than other treatments (about 7 g·kg^−1^ for both PLANT and MAP). As for glucose, at 28 days of storage was higher in MAP than in AIR, confirming a lower metabolism in MAP grapes probably associated with the presence of CO_2_. These results are confirmed by the soluble solids content that report a slight decrease during the storage without significant difference between treatments.

For all treatments and in both vineyards, an increase of phenolic content was also observed (much lower for grapes stored in MAP) immediately after 8 days of storage and up 21 days, followed by a reduction at the end storage. In Figure 4, data refer to 14-year-old vines, where after 28 days, grapes stored in AIR and on PLANT showed a higher phenolic content (1.92 and 1.80 g·kg^−1^, respectively) than the grapes stored in MAP (0.81 g·kg^−1^), but the same trend was observed also in the youngest vineyard. Probably, these results are associated with the biosynthesis of phenols, which is inhibited during postharvest storage in fruit and vegetables treated with elevated CO_2_ concentrations [34].

Regarding sensorial attributes, changes in flavor and presence of fizzy taste over time, are shown in Figure 3. It is important to notice that samples stored in MAP at the end of storage received the lowest flavor score, possibly related to the accumulation of CO_2_ in the cell sap in the form of carbonic acid which is the cause of the increase of the fizzy taste score, reported in figure for grapes from 14-year-old vines. On the other hand, it is important to highlight that the fizzy taste can also be due to fermentative processes caused by excessive accumulation of carbon dioxide and oxygen depletion typical of MAP [23] and associated with the accumulation of volatile compounds (ethyl acetate and ethanol). The latter aspect is more critical since when due to carbonic acid; it disappear after a few hours, with the evaporation of CO_2_.

**Figure 5:**
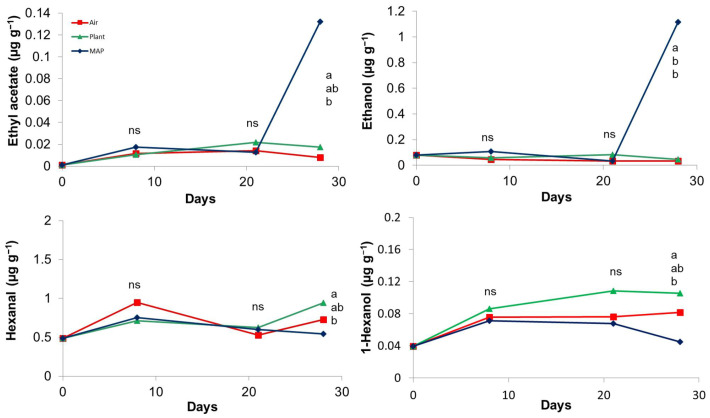
Effect of the storage treatment on the contents of ethyl acetate (**A**), ethanol (**B**), hexanal (**C**) (E-2-hexenal showed a similar trend), 1-hexanol (**D**), in grapes from 14-year-old vines (grapes from 4-year-old vines showed a similar trend). At each sampling different letters indicate significantly different mean values (*p* < 0.05 and Tukey test; ns: not significant).

With regard to aroma compounds a total of 21 volatiles, including six aldehydes (3-methylbutanal, pentanal, Z-2-butenal, hexanal, E-2-hexenal), six alcohols (ethanol, 1-hexanol, Z-3-hexen-1-ol, E-3-hexen-1-ol, 2-methyl-3-buten-2-ol, 1-pentanol), one ester (ethylacetate), four terpenes (D-limonene, cis-, and trans-linaloloxide, linalool), three ketones (3-penten-2-one, 1-penten-3-one, 6-methyl-5-hepten-2-one) one furan derivative (2-ethylfuran), one acid (acetic acid), were found in both grapes from 14-year old vines and 4-year old vines. Grapes from 14-year-old vines also showed the presence of E-2-butenal. For both grapes from 14-year-old vines and 4-year-old vines, most of the volatiles were not affected by the storage treatment except for ethyl acetate and ethanol accumulating in MAP and some typical compounds better preserved in plant. Particularly ethyl acetate and ethanol showed the same trend during the storage, with significant differences only at 28 days of storage, in which the highest concentrations were found in the treatment with MAP (Figure 5). In general, the increase in ethyl acetate and ethanol concentrations has been also reported in the headspace of apples and strawberries stored in high CO_2_ due to the fermentative metabolism [35,36]. Also in this trial, the ethanol and ethyl acetate could be used as indicators to determine the grade of degradation of table grapes.

On the other side, for both grapes from 14-year-old vines and 4-year-old vines, grapes kept on PLANT showed the highest concentration of E-2-hexenal, hexanal, and 1-hexanol, at the end of the storage (Figure 5), showing that grapes on the vine better maintained some typical compounds. The contents of all the other compounds, including e.g., the linalool, which is known to give floral notes, were not significantly different among the treatments.

Therefore, we can affirm that the MA packaging showed some advantages during the first 21 days of storage, as showed by the sensory evaluation of firmness, aroma, cluster, and stalk appearance, while at the end of storage the MA treatment suffered an accelerated process of fermentation likely characterized by the high content of ethanol and ethyl acetate, inducing the perception of fizzy taste.

## 4. Conclusions

Results of this experiment demonstrated that holding “Italia” table grapes on the PLANT, allowed a good preservation of the quality attributes (phenolic content, and flavor) compared to harvested product. Some additional benefits on cluster appearance score could be obtained by using MAP conditions, particularly if the grapes come from old vines, being more perishable than fruit from younger vine. Most volatile compounds did not change their concentration with the storage treatment, but ethyl acetate ant ethanol increased in MAP stored grapes at 28 days of storage, suggesting the occurrence of fermentation processes confirmed by a highest perception of fizzy taste in grape stored with MAP conditions. Nonetheless, 21 days are a very reasonable time for the commercialization of this product, considering that normally for packaged fresh produce a shelf-life of 7–12 days is normally accepted. Therefore, depending on the market and distribution needs and to the age of the vineyard, different storage strategies may be applied.

## Figures and Tables

**Table 1:** Results of the 2-way ANOVA for treatment (TR) and time of storage (T) a on quality attributes of “Italia” table grapes from a 4-year and 14-year-old vineyard. Within each row, each factor and their interaction have a significant effect for *p* ≤ 0.05 (*); *p* ≤ 0.01 (**); *p* ≤ 0.001 (***); *p* ≤ 0.0001 (****), or not significant (ns).

Quality Attributes	4-Year Old Vine			14-Year Old Vine		
	Treatment (TR)	Time (T)	TR X T	Treatment (TR)	Time (T)	TR X T
Firmness (N)	****	ns	**	**	**	***
Weight loss (%) ^1^	**	ns	ns	***	ns	ns
Hue angle (°)	ns	ns	ns	***	****	ns
Chroma	*	ns	*	****	*	*
TSS (%)	ns	ns	ns	ns	****	ns
pH-value	ns	ns	ns	ns	****	**
Titratable acidity (%)	ns	ns	ns	*	**	*
Phenols (g kg^−1^)	ns	ns	ns	*	ns	ns
Antioxidant activity (g kg^−1^)	ns	ns	ns	*	ns	ns
Acetaldheyde (nmole/g)	***	****	**	**	****	**
Ethanol (nmole/g)	*	****	ns	**	****	**
Tartaric acid (g kg^−1^)	ns	*	ns	ns	**	ns
Malic acid (g kg^−1^)	ns	ns	ns	ns	*	ns
Fumaric acid (g kg^−1^)	ns	ns	ns	ns	ns	ns
Citric acid (g kg^−1^)	ns	ns	ns	*	**	*
Succinic acid (g kg^−1^)	***	ns	ns	ns	*	ns
Sucrose (g kg^−1^)	ns	*	ns	ns	****	****
Glusose (g kg^−1^)	ns	ns	ns	ns	****	ns
Fructose (g kg^−1^)	ns	ns	ns	ns	*	ns
Sensorial Score (1 to 5)						
Cluster appearance score	****	****	****	****	****	****
Stalk appearance score	****	****	****	****	****	****
Berry appearance score	****	****	*	*	****	ns
Crunchiness	****	**	**	****	***	ns
Berry detachment	****	***	ns	****	****	****
Aroma	***	****	ns	*	****	**
Flavor	**	****	**	***	****	**
Sweetness	***	****	****	ns	****	**
Sourness	**	****	ns	**	****	ns
Fizzy taste	***	ns	ns	****	ns	ns
Overall evaluation	****	****	*	**	****	ns

^1^ AIR and MAP treatment.
